# Hierarchical phosphorylation of apical membrane antigen 1 is required for efficient red blood cell invasion by malaria parasites

**DOI:** 10.1038/srep34479

**Published:** 2016-10-04

**Authors:** Boris Prinz, Katherine L. Harvey, Louisa Wilcke, Ulrike Ruch, Klemens Engelberg, Laura Biller, Isabelle Lucet, Steffen Erkelenz, Dorothee Heincke, Tobias Spielmann, Christian Doerig, Conrad Kunick, Brendan S. Crabb, Paul R. Gilson, Tim W. Gilberger

**Affiliations:** 1 Bernhard-Nocht Institute for Tropical Medicine, 20359 Hamburg, Germany; 2 Centre for Biomedical Research, Burnet Institute, Melbourne, Victoria 3004, Australia; 3 Department of Microbiology and Immunology, University of Melbourne, Melbourne, Victoria 3010, Australia; 4 Department of Pathology and Molecular Medicine, M.G. DeGroote Institute for Infectious Disease Research, McMaster University, Hamilton, ON L8N 3Z5, Canada; 5 Chemical Biology Division, The Walter and Eliza Hall Institute, Melbourne, Victoria 3052, Australia; 6 Department of Microbiology, Monash University, Victoria 3800, Australia; 7 Institut für Medizinische und Pharmazeutische Chemie, Technische Universität Braunschweig, Beethovenstraße 55, 38106 Braunschweig, Germany; 8 Centre for Structural Systems Biology, 22607 Hamburg, Germany

## Abstract

Central to the pathogenesis of malaria is the proliferation of *Plasmodium falciparum* parasites within human erythrocytes. Parasites invade erythrocytes via a coordinated sequence of receptor-ligand interactions between the parasite and host cell. One key ligand, Apical Membrane Antigen 1 (AMA1), is a leading blood-stage vaccine and previous work indicates that phosphorylation of its cytoplasmic domain (CPD) is important to its function during invasion. Here we investigate the significance of each of the six available phospho-sites in the CPD. We confirm that the cyclic AMP/protein kinase A (PKA) signalling pathway elicits a phospho-priming step upon serine 610 (S_610_), which enables subsequent phosphorylation *in vitro* of a conserved, downstream threonine residue (T_613_) by glycogen synthase kinase 3 (GSK3). Both phosphorylation steps are required for AMA1 to function efficiently during invasion. This provides the first evidence that the functions of key invasion ligands of the malaria parasite are regulated by sequential phosphorylation steps.

*Plasmodium falciparum*, the causative agent of the most severe form of human malaria, remains an outstanding global health and socioeconomic burden causing more than 200 million clinical cases of disease and over 400,000 deaths per year^1^.

Throughout the erythrocytic life cycle, where the clinical manifestations of malaria arise, parasites remain largely protected intracellularly. ‘Merozoites’ produced after each cycle emerge and actively invade new erythrocytes with remarkable coordination and speed^2^,^3^. This requires the utiliy of highly specialized machinery to coordinate the complex sequence of receptor-ligand interactions and signal transduction events required for invasion (reviewed in refs 4 and 5).

One key player within this invasion machinery is the widely studied invasion ligand Apical Membrane Antigen 1 (AMA1). AMA1 is synthesized during schizogony as an 83 kDa precursor, which is cleaved within specialized secretory organelles called micronemes to yield a 66 kDa mature protein that then is trafficked to the merozoite surface, where it is anchored by a single transmembrane domain exposing a large N-terminal ectodomain and a short C-terminal cytoplasmic domain (CPD)^5^,^6^. Several studies have shown that the extracellular domain of AMA1 binds the host cell via a parasite-derived RON (rhoptry neck protein) complex, which is translocated prior to invasion^7^,^8^,^9^,^10^,^11^,^12^,^13^,^14^. This interaction elicits formation of a cell-cell membrane junction, which is thought to provide an intimate physical link between the host and parasite and serve as a stable anchoring structure upon which the merozoite can apply traction as it invades (reviewed in ref. 15). In addition to important interactions provided by the AMA1 ectodomain, the highly conserved cytoplasmic domain has been shown to be crucial for the function of AMA1 during invasion^3^. One important feature of the AMA1 CPD appears to be its phosphorylation at S_610_ by cyclic AMP-dependent protein kinase A (PKA)^16^, among other residues that are phosphorylated with unknown functional implications^3^,^16^,^17^,^18^. Protein phosphorylation is a universal intracellular regulatory mechanism that has been described for many apicomplexan proteins (reviewed in ref. 19). The functional regulation of invasion ligands by phosphorylation, such as that described for reticulocyte binding-like protein 4 (Rh4), would be an elegant mechanism to help coordinate the invasion process^20^.

Here we reveal key functional phosphorylation events upon AMA1, confirming the primary role of PKA in initiating a hierarchy of phosphorylation events involving a secondary kinase glycogen synthase kinase 3 (GSK3). Multiple phosphorylation appears to impart functional efficiency onto AMA1 during erythrocyte invasion by *P. falciparum.*

## Results

### Identification of functional phosphorylation sites

**Figure 1:**
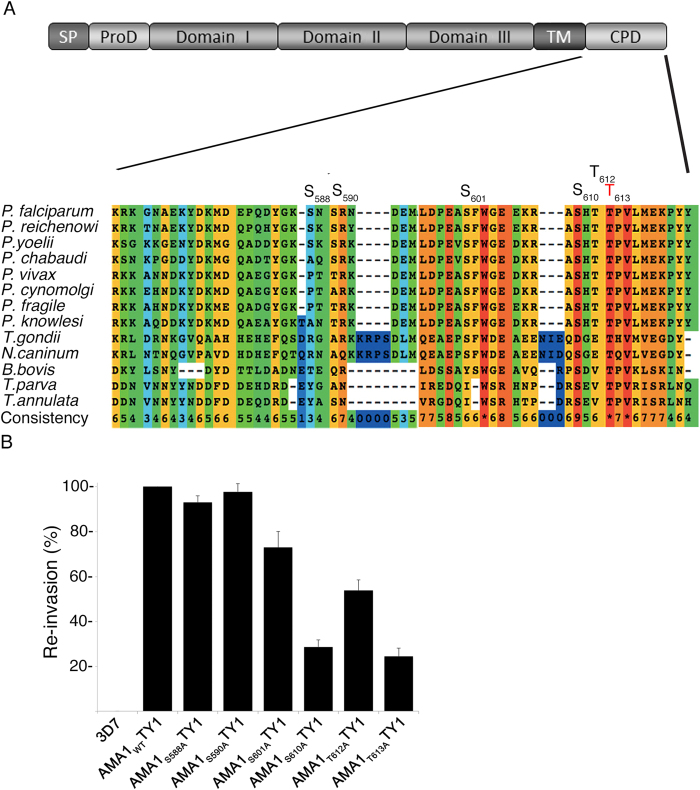
Functional analysis of AMA1 phosphorylation sites. (**A**) Multiple alignment of the cytoplasmic domain of various AMA1 from Apicomplexa. The conservation is scored and colour coded by PRALINE (www.ibi.vu.nl). Amino acids predicted to be phosphorylated in *P. falciparum* by NetPhos (www.cbs.dtu.dk/services/NetPhos) and confirmed by mass spectrometry^17^,^18^,^21^ are highlighted. S_610_ was previously shown to be essential for efficient erythrocyte invasion. (**B**) The invasion ability of the different AMA1-TY1 parasite strains expressing AMA1 with single mutations in each phosphorylation site was investigated by an invasion assay. Assays were performed in the presence of 100 μg/mL R1 peptide. Re-invasion was normalised to 3D7 and AMA1_WT_-TY1, which were used as controls. Error bars correspond to standard errors. Assays were performed in triplicates in three independent experiments.

Six potential phosphorylation sites are present within the cytoplasmic domain of AMA1, S_588_, S_590_, S_601_, S_610_, T_612_, and T_613_, and phosphoproteomics on parasite material indicates that all of these are indeed phosphorylated^17^,^18^,^21^ (Fig. 1A). While the importance of S_610_ for red blood cell invasion has been previously reported^16^, the functional significance of the other phosphorylated residues is unknown. We used a complementation approach as previously described by Treeck *et al*.^3^ to determine the necessity for each of these residues. Briefly, full-length AMA1 derived from the W2mef parasite strain bearing phosphorylation-defective mutations (S_588_A, S_590_A, S_601_A, S_610_A, T_612_A, T_613_A) were ectopically expressed as TY1 fusion proteins in the 3D7 parasite cell line. Importantly, the ectodomain of W2mef AMA1 contains certain amino acid changes that render it resistant to the invasion blocking effects of R1 peptide, which binds 3D7 AMA1 and inhibits RON binding and junction formation^22^. Thus, to test the invasive capacity of each of the mutants, transgenic 3D7 merozoites were allowed to invade red blood cells in the presence of R1 peptide to exclude function of the endogenous protein. As anticipated, over-expression of the wild-type protein from the W2mef strain (AMA1_WT_-TY1) functionally complemented the endogenous protein and, as previously reported^16^, mutation of S_610_ (AMA1_S610A_-TY1) decreased invasion efficiency by approximately 70% (Fig. 1B). Interestingly, a comparable reduction of invasion efficiency was observed for the T_613_ mutant (AMA1_T613A_-TY1). This was in contrast to the AMA1_S588A_-TY1 and AMA1_S590A_-TY1 mutant, whose complementation capacities were barely reduced, and the AMA1_S601A_-TY1 and AMA1_T612A_-TY1 mutants, which displayed intermediate reduction phenotypes (Fig. 1B). This result is unsurprising given the high level of conservation of S_610_ and T_613_ across the phylum Apicomplexa: S_610_ is conserved in all species of *Plasmodium*, *Neospora*, *Theileria and Babesia* (in *T. gondii* an aspartic acid residue can act as a phosphomimetic), and T_613_ is absolutely conserved, indicating a key function of these two residues.

### S_610_ is targeted by PKA

**Figure 2:**
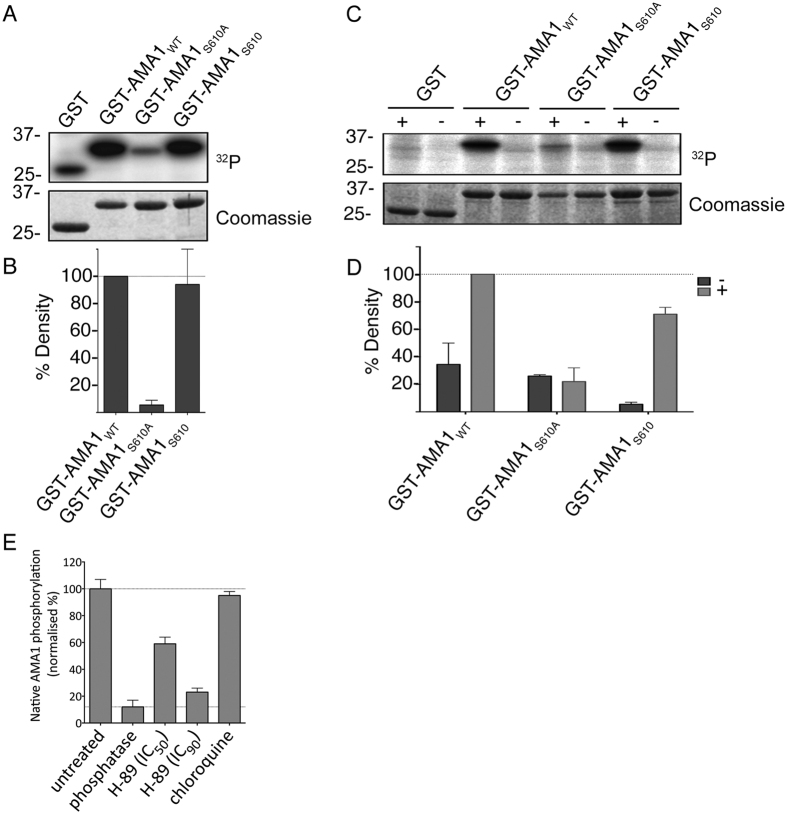
S_610_ is targeted by *Pf*PKA. (**A)** Autoradiograph and SDS-PAGE of recombinant AMA1-GST variants treated with bovine heart PKA in the presence of cAMP and ^32^P-γ-ATP. The AMA1 variants either had S610 mutated to alanine (AMA1_S610A_) or had all phospho-sites except for S610 (AMA1_S610_) mutated. Purified GST serves as negative and AMA1_WT_ as positive control. (**B)** Signal intensities were quantified using Image Gauge software (Fujifilm, Image Gauge V4.0). Signal intensity of the GST sample was subtracted from each sample and they were then normalized against the signal intensity of the AMA1_WT_ sample. Error bars correspond to standard deviation of two independent experiments done in triplicate. (**C)** SDS-PAGE and autoradiograph of *in vitro* phosphorylation of GST, AMA1_WT_, AMA1_S610A_ and AMA1_S610_ after incubation with schizont material in the presence of ^32^P-γ-ATP and either with (+) or without (-) cAMP. (**D)** Densitometric quantification with error bars corresponds to standard deviation of two independent experiments done in triplicates. (**E**) Sandwich ELISA demonstrating H-89-induced inhibition of native AMA1 phosphorylation at S_610_. Parasites were treated with H-89 for 2 hours during egress and invasion and a mouse anti-*Pf*AMA1 antibody was used to capture *Pf*AMA1 from culture lysates. Phosphorylation of S_610_ was detected in an ELISA format using the anti-*Pf*AMA1S_610_p antibody. Histograms were generated after normalizing against uninfected (0%, background) and untreated (100%) culture signals. Lambda phosphatase-treatment was used to denote zero phosphorylation and chloroquine treatment was used to exclude parasite growth arrest as a cause for reduced phosphorylation.

While extensive data by Leykauf *et al*.^16^ indicated that PKA targets AMA1_S610_, the evidence is based on the observation that mutation of S_610_ precludes phosphorylation and is therefore largely indirect. To first demonstrate that PKA phosphorylates S_610_ *in vitro*, recombinant AMA1 CPD variants were generated as GST fusion proteins for *in vitro* phosphorylation assays. Wild-type AMA1 (GST-AMA1_WT_) and phosphorylation-defective S_610_A (AMA1_S610A_) were compared with AMA1 with a single acceptor site at S_610_ left intact and all other phosphorylation sites mutated to non-phosphorylatable alanines (AMA1_S610_). Phosphorylation of AMA1_S610A_ by purified bovine PKA (Fig. 2A,B) or by parasite extracts stimulated with cyclic AMP (Fig. 2C,D) was drastically reduced compared to AMA1_WT_, while AMA1_S610_ was phosphorylated to comparable levels as AMA1_WT_. This indicates that PKA is responsible for S_610_ phosphorylation *in vitro*. To then probe into PKA targets AMA1 at S_610_ *in vivo*, parasites were treated with the PKA inhibitor H-89 for 2 hours. Subsequently, a “sandwich” ELISA was performed to purify native AMA1 from the parasites, using an antibody against the ectodomain, and the resultant levels of phosphorylation of S_610_ were captured using an anti-phospho AMA1_S610_ antibody. At 4.9 μM and 33.1 μM (IC_50_ and IC_90_ as determined over a 2 hour treatment period; data not shown), H-89 inhibited phosphorylation by 47% and 87%, when considering the Lambda phosphatase dephosphorylated sample as having a baseline signal (Fig. 2E). This further supports the role of *Pf*PKA in AMA1 phosphorylation at S_610_ *in P. falciparum* parasites.

### T_613_ is phosphorylated by GSK3 *in vitro*

**Figure 3:**
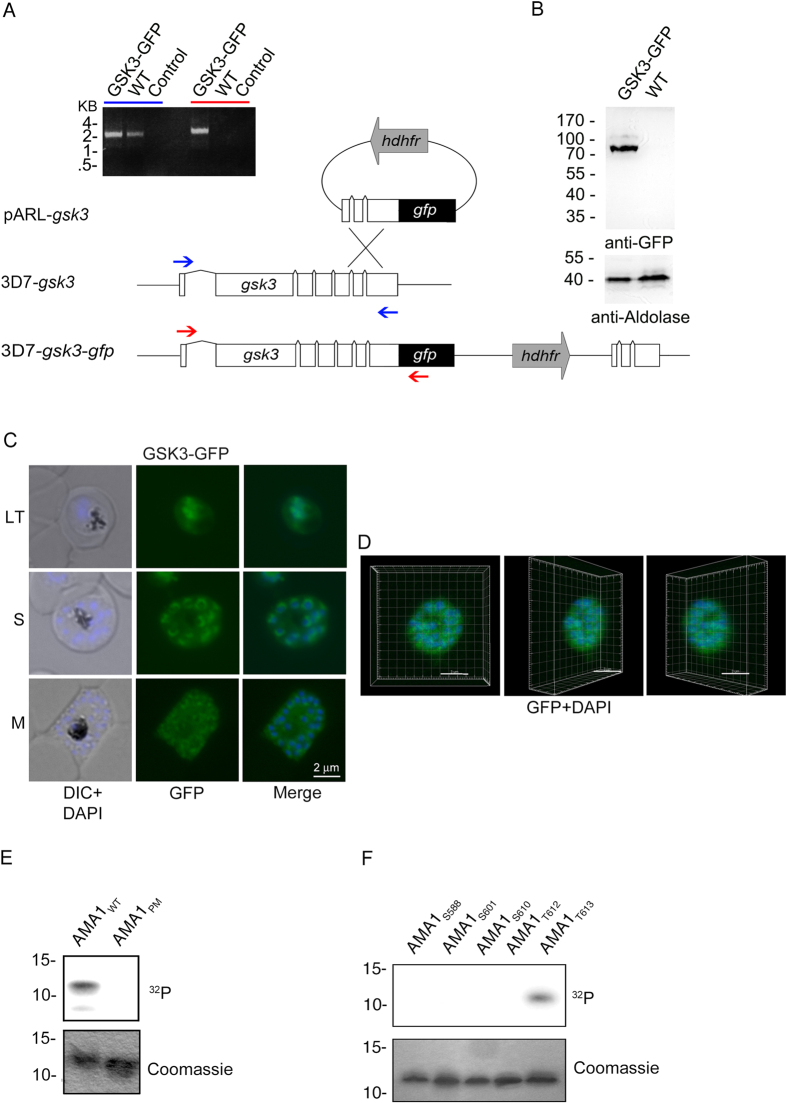
T_613_ is phosphorylated by GSK3. (**A–D**) Expression and localization of *Pf*GSK3 and its PKA dependent phosphorylation of AMA1. (**A**) Schematic drawing of the GSK3-GFP 3′replacement approach in 3D7 parasites and diagnostic PCR revealing plasmid integration. The *gsk3* gene has a six exon structure and an open reading frame of 2472 base pairs. Approximately 1 kb of the 3′ end was fused with the coding sequence of GFP (black) and cloned into a pARL derivate (pARL-gsk3-3’repl-gfp). The human dihydrofolate reductase (hDHFR, grey box) of the plasmid allowed selection of transgenic parasites. Position of oligonucleotides used for diagnostic PCR are shown with blue and red arrows. Sizes are indicated in kilo bases (kb). (**B**) Expression of *Pf*GSK3-GFP in late stage parasites was analyzed by Western blot analysis using anti-GFP specific antibodies. Anti-Aldolase specific antibodies were used as a loading control. (**C**,**D**) Epifluorescence (**C**) and confocal (**D**) localization of *Pf*GSK3-GFP in late trophozoites (LT) schizonts (S) and merozoites (M) revealed perinuclear and cytosolic distribution. Nuclei are stained with DAPI (blue). Scale bars, 2 μm. **E.** SDS-PAGE and autoradiograph of *in vitro* phosphorylation samples (upper panel) as well as coomassie stained loading (lower panel) of AMA1_WT_ and AMA1_PM_ incubated with human GSK3β (hGSK3β). (**F**) Differential *in vitro* phosphorylation of AMA1 variants with single phosphorylation sites (AMA1_S588_, AMA1_S601_, AMA1_S610_, AMA1_T612_, AMA1_T613_) by hGSK3β. SDS-PAGE and autoradiograph of the *in vitro* phosphorylation samples (upper panel) as well as coomassie stained loading (lower panel) are shown.

Based on NetPhosK predictive software, T_613_ is a putative recognition site for proline-directed kinases such as p38MAPK, cdk5 and glycogen synthase kinase 3 (GSK3). There are two MAP kinases in the *P. falciparum* genome *Pfmap1* and *Pfmap2* that are expressed in blood stages. The latter kinase is essential and might be required during schizogony as well as for other life cycle stages^23^. *P. falciparum* also has a cdk5 homolog called protein kinase 5 which appears to have nuclear functions^24^. A GSK3 homologue (*Pf*GSK3) is also encoded in the parasite genome and appears to be constitutively expressed throughout the asexual cell cycle based on RNA expression and antibody based protein detection^25^,^26^,^27^,^28^. GSK3 is known to typically recognize pre-phosphorylated sequences^29^, a particular feature that could explain the pivotal role of S_610_ phosphorylation. This characteristic suggests that *Pf*GSK3 could be involved in T_613_ phosphorylation subsequent to phosphorylation of S_610_ by PKA. To confirm *Pf*GSK3 expression in blood stages, the endogenous gene (PF3D7_1316000) was tagged with green fluorescent protein (GFP) (Fig. 3A,B) and localization studies indicate that the kinase localizes in nuclear or perinuclear compartments as well as in the cytosol in erythrocytic stages of the parasite (Fig. 3C,D).

Thus, we examined phosphorylation of the AMA1 CPD by human GSK3β *in vitro* and found that, indeed, *h*GSK3β phosphorylated AMA1_WT_ but not AMA1_PM_ in which all the phospho-sites have been mutated (Fig. 3E). To determine the specific phospho-site we again used different AMA1 variants where only a single phospho-acceptor site was available (AMA1_S588_, AMA1_S601_, AMA1_S610_, AMA1_T612_, AMA1_T613_) and found that only AMA1_T613_ was phosphorylated by *h*GSK3β (Fig. 3F).

### S_610_ phosphorylation is a prerequisite for efficient T_613_ phosphorylation

**Figure 4:**
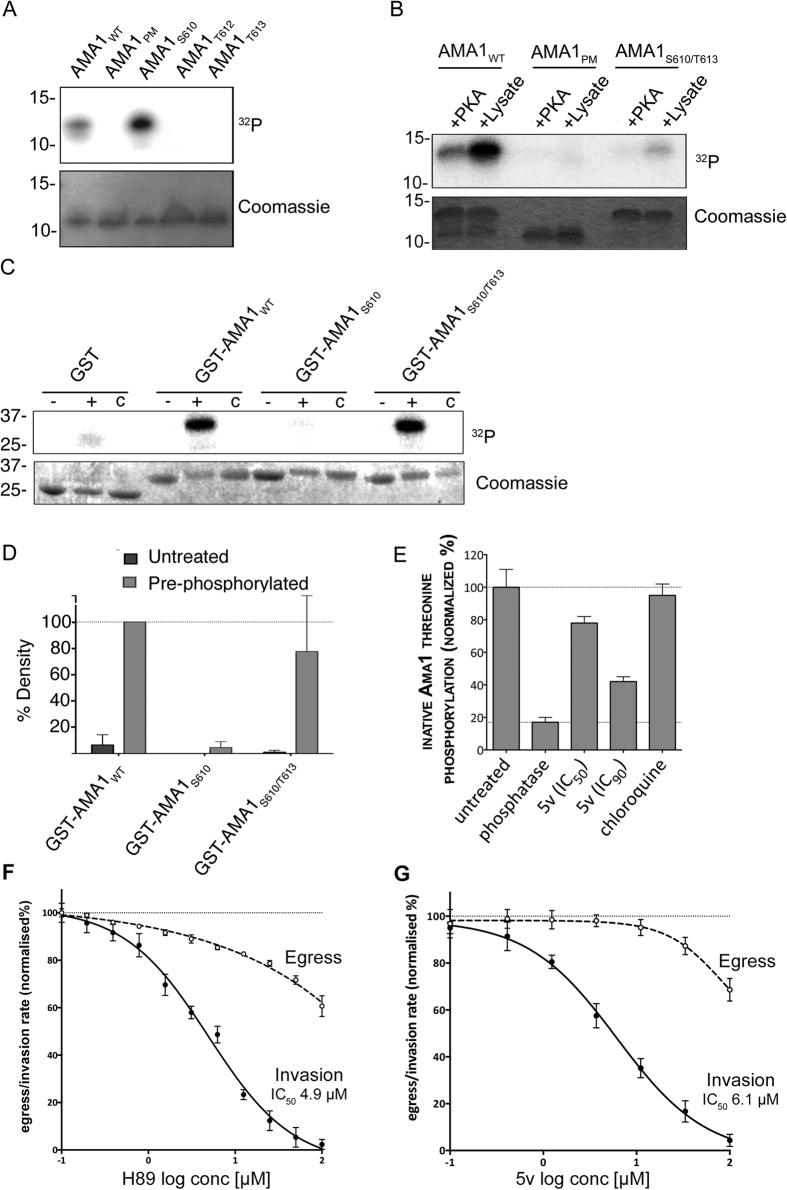
S_610_ phosphorylation is a prerequisite for efficient T_613_ is phosphorylation. (**A**) Autoradiograph of *in vitro* phosphorylation samples (upper panel) as well as coomassie stained loading (lower panel) are shown. AMA1 variants displaying either all (AMA1_WT_), none (AMA1_PM_) or only single phosphorylation sites (AMA1_S610_, AMA1_T612_, AMA1_T613_) were incubated with schizont material in the presence of cAMP and ^32^P-γ-ATP. (**B)** Phosphorylation of PKA pre-phosphorylated AMA1_WT_ and mutants AMA1_PM_ and AMA1_S610/T613_. Recombinant proteins were first incubated with PKA in the presence of non-labeled ATP. Subsequently, these pre-treated AMA1 were incubated with either PKA or a schizont extract in the presence of ^32^P-γ-ATP. AMA1_PM_ was used as a control. SDS-PAGE and radiograph of *in vitro* phosphorylation samples (upper panel) as well as coomassie stained loading (lower panel) are shown. (**C)** *In vitro* phosphorylation of AMA1-GST fusion variants (AMA1_WT_, AMA1_S610_, AMA1_S610/T613_) either as unphosphorylated (-) or PKA-pre-phosphorylated (+) substrates in the presence of recombinant *Pf*GSK3β and ^32^P-γ-ATP or without the addition of *Pf*GSK3β (c). (**D)** Signal intensities were quantified. Error bars correspond to standard deviation of two independent experiments done in triplicates. (**E)** Sandwich ELISA demonstrating 5v-induced inhibition of native AMA1 threonine phosphorylation. Parasites were treated with 5v and a rabbit anti-*Pf*AMA1 antibody was used to capture *Pf*AMA1 from culture lysates. Threonine phosphorylation was detected in an ELISA format using a mouse anti-phosphothreonine antibody. Histograms were generated after normalizing against uninfected (0%, background) and untreated (100%) culture signals. Phosphatase-treatment was used to denote zero phosphorylation and chloroquine treatment was used to exclude parasite growth arrest as a cause for reduced phosphorylation. (**F)** The *Pf*PKA inhibitor H89, weakly inhibited egress and strongly inhibited invasion in *P. falciparum* reporter parasites transfected with secreted Nanoluciferase. Luciferase activity in relative light units was measured and dose-response curves were plotted for egress (dashed line) and invasion (solid line) after normalizing against uninfected (0%) and untreated (100%) culture sample signals. Two biological replicates were performed in triplicate; error bars denote one standard deviation. (**G)** The *Pf*GSK inhibitor 5v strongly inhibits invasion. The experiment was performed as per F.

Because GSK3 typically targets pre-phosphorylated substrates, and because there is evidence to suggest that S_610_ acts as a master switch for downstream AMA1 phosphorylation^16^, we investigated the dynamics of S_610_ and T_613_ phosphorylation. The AMA1 CPD variants with single phosphorylation sites at S_610_ and T_613_ (AMA1_WT_, AMA1_S610_ and AMA1_T613_) were subjected to *in vitro* phosphorylation assays with parasite extracts, but, while AMA1_WT_ and AMA1_S610_ were phosphorylated, presumably by residual PKA, AMA1_T613_ displayed no signal (Fig. 4A). We therefore hypothesized that S_610_, and likely its phosphorylation, must be present to prime the phosphorylation of T_613_. To test this hypothesis we generated an AMA1 variant displaying only two phosphorylation sites, S_610_ and T_613_ (AMA1_S610/T613_). Recombinant proteins were first incubated with PKA in the presence of non-labeled ATP. Subsequently, these pre-treated AMA1 variants were incubated with either secondary PKA or parasite material in the presence of ^32^P-γ-ATP, and only incubation with the parasite material resulted in labeling on the substrate in AMA1_WT_ and AMA1_S610/T613_, but not in the negative control AMA1_PM_ (Fig. 4B). Some additional background phosphorylation by PKA of AMA1_WT_ was observed, although this is significantly less than the subsequent phosphorylation by parasite schizont material. The relative strong phosphorylation of the AMA1_WT_ in comparison to AMA1_S610/T613_ is explained by the presence of other phosphorylation sites such as S_588_ and S_590_ that are known to be phosphorylated *in vivo*^18^. It could also indicate that there are further phosphorylation events (for example T_612_) that also require phospho-priming. This suggests that phosphorylation by PKA is required for further phosphorylation of T_613_. We then demonstrated that pre-phosphorylation by PKA is required specifically for phosphorylation by recombinant *P. falciparum* GSK3 protein. After PKA phosphorylation of AMA1_WT_, AMA1_S610_ and AMA1_S610/T613_, only the latter variant could be phosphorylated by *Pf*GSK3β (Fig. 4C,D). These experiments show that recombinant GSK3 from Plasmodium parasites requires pre-phosphorylation of AMA1 for efficient substrate phosphorylation, while the human counterpart phosphorylates AMA1 without PKA-dependent pre-phosphorylation. This might be due to a lower *Pf*GSK3 concentration, but could also point towards different requirements for substrate recognition or phosphorylation between human and plasmodium GSK3.

Finally, to confirm that GSK3 indeed phosphorylates AMA1 inside parasites, a *Pf*GSK3 specific inhibitor was used. The 5v compound belonging to the 3-amino-4-arylthieno[2,3-*b*]pyridines was previously identified and characterized in a high-throughput screen showing selective inhibition of the plasmodial enzyme and blood stage growth of the parasite^30^. Therefore, 5v treated parasites were used in another “sandwich” ELISA. Threonine phosphorylation of the cytoplasmic domain of AMA1 was quantified using an anti-phosphothreonine antibody. Compound 5v caused a reduction in AMA1 threonine phosphorylation by 25% and 66% (when considering the phosphatase-treated sample as a baseline) at concentrations of 6.1 μM and 50.1 μM respectively (IC_50_ and IC_90_ as determined over a 2 hours treatment period; data not shown) (Fig. 4E). Taken together, these data indicate that functional phosphorylation of AMA1 occurs in a two-step manner whereby PKA initially targets S_610_ for subsequent phosphorylation of T_613_ by GSK3.

To chemically validate that phosphorylation of AMA1 promotes erythrocyte invasion, the two kinases that were now implicated in this process were inhibited to prevent phosphorylation. To observe the effect upon invasion, an assay was developed with transgenic parasites that express and secrete luciferase into the parasitophorous vacuole^31^. Synchronised parasites were allowed to egress and invade over a two hour period in the presence of PKA inhibitor H-89 or *Pf*GSK3 inhibitor 5v. Although H-89 probably has activity on other kinases we hoped to concentrate its effects against PKA by restricting its use to a two hour window when PKA was maximally expressed. A sample of the culture supernatant, which contained the secreted luciferase that is released during host cell rupture, was collected to quantify levels of egress. To measure invasion, the drug was washed from the cultures. Further invasions were blocked using heparin, and whole culture samples were collected 24h later during peak luciferase expression. Dose-response curves were generated for egress and invasion indicate that the *Pf*PKA inhibitor H-89^16^ had an inhibitory effect on egress at high concentrations, but also specifically inhibited invasion with an IC_50_ concentration of 4.9 μM (Fig. 4F). The *Pf*GSK3 inhibitor 5v performed similarly to H89 with a small effect on egress at concentrations greater than 10 μM and a stronger effect upon invasion with an IC_50_ concentration of 6.1 μM (Fig. 4G). The observation that both inhibitors have a drastic effect on the invasion capability of the parasite is consistent with the fact that both their target kinases are required for sequential AMA1 phosphorylation steps that are needed for invasion.

## Discussion

The invasion of erythrocytes by malaria parasites is a rapid but highly complex process, for which the specific molecular details have been only partially resolved. Several post-translational modifications of the parasite’s invasion machinery, including protein phosphorylation, are thought to act as important checkpoints to facilitate efficient entry into the host cell^17^. It was previously reported that the AMA1 cytoplasmic domain is extensively phosphorylated, and emerging evidence suggests that certain phosphorylation events play a central role in the function of this protein during invasion^3^,^16^,^17^,^18^. This was reiterated by recent findings, demonstrating that the cytoplasmic moiety of the reticulocyte binding-like protein 4 (*Pf*Rh4) needs to be phosphorylated in order to achieve efficient completion of the invasion process^20^.

In this study, we used an *in vivo* screen to analyse the contribution of each CPD phospho-site toward the function of AMA1, confirming the importance of S_610_ and further identifying T_613_ as a key residue for efficient invasion. We were able to confirm the role of protein kinase A (PKA) in phosphorylation of S_610_ and revealed GSK3 as a strong candidate for phosphorylation of T_613_. This was further substantiated by the use of specific inhibitors for *Pf*PKA and *Pf*GSK3 that were able to block invasion. These kinases probably phosphorylate many proteins and at least the H89 inhibitor targets other kinases but by limiting their application to a two hours invasion window we hoped to restrict their major effects to AMA1. Eliminating the possible roles of other proline-directed kinases such as *Pf*MAP2 kinases and *Pf*PK5 would also help validate *Pf*GSK3 as the kinase acting upon T_613_. Importantly, we found that T_613_ phosphorylation by parasite lysates and by recombinant GSK3 is dependent upon phosphorylation of S_610_ by PKA, which is consistent with the documented preference of GSK3 for pre-phosphorylated substrates^29^ and the indication that S_610_ acts as a master switch for phosphorylation.

PKA is a ubiquitous kinase with numerous roles across many different organisms, and several studies using stimulants and inhibitors of PKA and modulation of gene expression have highlighted the indispensability of this kinase for parasite growth^21^,^32^,^33^,^34^. *Pf*PKA is expressed late in the asexual cell cycle^25^,^27^ and a recent study identified hundreds of merozoite phosphoproteins with PKA phosphorylation motifs, indicating that this kinase likely has multiple important functions during merozoite development and/or invasion in addition to phosphorylation of AMA1^28^. The role of GSK3 in parasites remains relatively uncharacterized and AMA1 is the first known *in vitro* substrate of the parasite kinase. Previous findings using cross-reacting antibodies directed against mammalian GSK3 indicate that *Pf*GSK3 associates with Maurer’s clefts in the iRBC cytosol and it was assumed that it might function in cytoskeletal stability or controlling ion channels for the circadian rhythm^35^. While export of some *Pf*GSK3 cannot be excluded, our data, which are based on GFP-tagged *Pf*GSK3 expressed from the cognate locus, show that the vast majority remains in the parasite either as a nuclear-associated or cytosolic protein. The cytosolic fraction of *Pf*GSK3 is consistent with its role in AMA1 phospho-activation in late stage parasites. Additional nuclear localization of GSK3 is also described for other cell types^36^,^37^,^38^ and is a reflection of the broad range of functions of these kinases that includes the regulation of cell migration and neurogenesis^39^,^40^. Our imaging data suggest that such a multiplicity of functions is also shared by the *Plasmodium* GSK3.

Since GSK3 appears to be constitutively expressed with no evident regulation mechanisms, this presents a sophisticated means of controlling AMA1 phosphorylation. PKA signaling, on the contrary, has multiple upstream regulators (described below). Whether phosphorylation of S_610_ itself has a functional role in addition to acting to control T_613_ phosphorylation is yet to be determined. While GSK3 generally responds to phospho-priming, S_610_ is not located within the GSK3 recognition sequence and thus its phosphorylation may not be a classical phospho-priming step but rather act by opening the conformation of the AMA1 CPD to expose T_613_ for phosphorylation. Alternatively, the priming mechanism may be conserved but the location of the priming site may have diverged between the mammalian and *Plasmodium* enzymes. Integrating these findings, we propose the following scenario for activation of AMA1: During schizogony the activity of a bicarbonate-sensitive cytoplasmic adenylyl cyclase increases, which generates the cyclic AMP necessary to activate PKA by dissociation of the active catalytic subunit from the regulatory subunit^41^. PKA phosphorylation of AMA1 at S_610_ likely defines the rate-limiting step in the hierarchical AMA1 phosphorylation cascade, where phosphorylation of T_613_, likely by GSK3, follows.

The kinase(s) that could phosphorylate the other sites present on the CPD, such as S_588_, S_590_, S_601_, and T_612_ are not known but individually they do not seem to be as functionally important for invasion since phosphorylation-defective mutations only moderately reduce invasion efficiency. Interestingly, phosphorylation of S_610_ is likely also important for the phosphorylation of these sites as it is hypothesized to be a master switch for all AMA1 phosphorylation^16^.

The functional specific steps that follow AMA1 phosphorylation are not presently understood. AMA1 appears to be phosphorylated at multiple sites soon after synthesis in late schizonts, as the 83 kDa proprotein form is phosphorylated alongside the processed 66 kDa form, but the purpose of this is currently unknown^16^. However, phosphorylation appears to be necessary during moving junction formation because that event was captured in the complementation assays using R1 peptide. Live cell imaging of AMA1_S610A_ parasites in this complementation assay showed binding and vigorous deformation of the host erythrocyte despite the inability to invade^16^.

We originally proposed that phosphorylation may facilitate binding of the AMA1 CPD to the actomyosin invasion motor via an aldolase bridge thereby permitting the motor to apply traction to the junction and facilitating merozoite entry into the erythrocyte^16^. However, phosphorylation does not appear to be necessary for AMA1 binding to rabbit aldolase *in vitro* and the binding of ligands to aldolase does not even appear to be necessary for invasion in *Toxoplasma gondii*^42^. But another study demonstrated that the CPDs of some *P. falciparum* ligands promote actin polymerization likely via aldolase, which also binds and polymerises actin for actin-myosin motor function, however AMA1 was not tested^43^. To determine whether the CPD of AMA1 interacts with any proteins in a phosphorylation-dependent manner will certainly aid the resolution of the function of this signal cascade.

The basic structure of AMA1 and the fact that it does not need to be highly expressed for invasion^44^ argues that phosphorylation may act as a signal transducer where the AMA1 ectodomain can detect changes in the micro-environment, such as RON binding and junction formation, and modulate the cytoplasmic region to transfer signals to the intracellular environment. In this sense, phospho-signaling could occur in response to junction formation as a checkpoint to trigger the subsequent steps in the invasion process. The initiation of invasion events that follow RON binding, such as internalisation, organelle discharge and ligand cleavage, take place in the presence of AMA1-binding peptides that mimic this binding event^3^,^45^,^46^. However, a difficulty with this model is that the AMA1 CPD is already partially phosphorylated prior to invasion^16^. Perhaps AMA1 becomes fully phosphorylated in response to RON complex binding, or alternatively it could become dephosphorylated - the function of the calcium dependent phosphatase calcineurin has recently been shown to be required for invasion^47^,^48^. S_610_ could become rapidly dephosphorylated following T_613_ phosphorylation if it serves only as a master switch, yielding a singly phosphorylated substrate. Or phosphorylation and dephosphorylation could occur continuously throughout invasion. The dynamics and functional consequences of S_610_/T_613_ phosphorylation will be explored further in future studies.

Since AMA1 was first recognised as a central invasion ligand 15 years ago^49^, efforts are ongoing to reveal the ultimate role of this protein. Our study may have important biological significance revealing the first known hierarchical phosphorylation events that lead to activation of downstream events during invasion of host cells by malaria parasites. Furthermore, it has become increasingly clear that a greater understanding of the molecular aspects of invasion is vital to inform the rational identification of future vaccine and drug targets, and such insights are likely to open many avenues for the development of novel interventions against malaria and other Apicomplexan diseases.

## Methods

### Nucleic acids and DNA constructs

The *ama1* gene (PlasmoDB accession number PF3D7_1133400) was amplified from W2mef *P. falciparum* cDNA. *In vitro* mutagenesis of *ama1* was achieved either by the reverse oligonucleotides containing the mutation site (stop-codon was removed) or by using a two-step primer directed PCR mutagenesis method^50^ with Phusion^®^ High-Fidelity DNA Polymerase (NEB). To ensure correct timing of gene transcription, the *ama1* transgenes were controlled by the *ama1*-promotor using the pARL1-Vector^51^ with a C-terminal TY1 tag. Constructs were cloned via *KpnI/AvrII* restriction sites into the transfection vector.

For the generation of GST (glutathione-S-transferase) fusion proteins the DNA sequence of the *Pf*AMA1 C-terminal tail was cloned via *BamHI/XhoI* restriction sites and without a stop-codon into the bacterial expression vector pGEX-4T-1 (GE Healthcare). This construct produces fusion proteins of *Pf*AMA1 tail N-terminally bound to GST. Different mutants of the GST-*Pf*AMA1 tail fusion protein were achieved by site-directed mutagenesis. Purification of GST fusion proteins was performed according to the manufacturer’s protocol (GE-Healthcare).

3D7 parasites expressing *Pf*GSK3 as GFP fusion protein were generated by a 3’replacement of the endogenous *gsk3* gene. The last 854 bp of the *gsk3* coding region (PlasmoDB accession number PF3D7_0321400) was amplified from 3D7 gDNA without the stop-codon and transferred into the transfection vector pARL-GFP via the *NotI/AvrII* restriction sites. By using *NotI* restriction site the *ama1* promotor was removed which leads, only after integration, to a detectable GSK3-GFP construct. All constructs were confirmed by sequencing. Integration at the right locus was confirmed by PCR analysis of the gDNA.

### Immuno-Blots and antisera

The probes were collected from ~7–8% synchronized schizont cultures. The parasite material was suspended in SDS sample buffer and incubated at 90 °C for 5 minutes. Proteins were separated on 10–15% SDS-PAGE and subsequently transferred onto a nitrocellulose membrane. Immunoblots were performed using standard procedures and developed by chemiluminescence using ECL (GE Healthcare). Antibodies used in immunodetection were rabbit monoclonal anti-TY1 (Diagenode) and monoclonal anti-GFP (Roche). Anti-TY1 was used 1:1000, anti-GFP antibody 1:1000 and anti-Aldolase 1:5000 diluted in phosphate-buffered saline (PBS) with 5% w/v skim milk. Secondary antibodies were sheep anti-rabbit IgG horseradish peroxidase (Sigma) and sheep anti-mouse IgG horseradish peroxidase (Roche) used 1:5000 diluted in PBS with 5% w/v skim milk.

### Parasite culture and transfection

*P. falciparum* asexual blood stages were cultured in 0^+^ erythrocytes. W2mef were derived from the Indochina III/CDC strain. 3D7 and W2mef were transfected with 100 μg of purified plasmid DNA as described previously^52^. Positive selection of transfected parasites was achieved by addition of 10 nM WR99210, an antifolate that selects for the presence of the human *dhfr* gene. For single cross-over integration of the *gsk3-gfp* into the endogenous gene locus, after transfection the parasites were alternately grown with and without WR99210 pressure (2–3 weeks for each off-drug interval) to promote integration.

### Erythrocyte invasion assay

Parasite erythrocyte invasion assays were performed using 3D7 and transgenic parasites. Parasitemia of sorbitol synchronized parasite culture was measured using a Becton-Dickinson LSR II flow cytometer (FACS). For the experiment a parasite culture with 0.5% parasitemia of trophozoites (4% hematocrit) was incubated in a 96-well Plate (150 μL per well) under standard culturing conditions for 48 hours to allow re-infection in the presence or absence (control) of 100 μg/mL R1. After reinvasion occurred, parasites were stained with 0,1 μM syto^®^16 green fluorescent nucleic acid stain (Invitrogen) for 30 minutes at 37 °C and washed three times with media. For proper cell counting each culture was diluted 1:10 in media and afterwards applied to the FACS for measuring parasitemia. Assays were performed in triplicates on three independent occasions.

### Luciferase based egress and invasion assays

H89 and 5v were serially diluted in DMSO at 500x working concentration to be added to luciferase-expressing parasite cultures with a final concentration of 0.2% DMSO. Synchronised late-stage parasite cultures (~44–48 hour post invasion) were seeded into 96-well microplates at 5% parasitaemia and 2% haematocrit. Compounds were titrated in triplicate and incubated over egress and invasion (37 °C, 2 h). Samples of culture supernatant were collected to determine the amount of luciferase released during egress, then the compounds were washed out to prevent inhibition of parasite growth and 100 μg/mL heparin sodium salt (Sigma) was added to prevent any further invasion events. Parasites were returned to culture for a further 24 h and whole culture samples were collected to determine the amount of luciferase expressing parasites present. Whole culture samples were mixed with 0.6x sample volume PBS, 0.4x sample volume Luciferase Cell Culture Lysis 5X Reagent (Promega) and 0.01x sample volume NanoGlo Luciferase Assay Substrate (Promega). Light units emitted from the reaction were measured in a luminometer with the gain reduced by 10% for the sample with the highest signal to prevent saturation. Relative light units were plotted in Prism 6 (GraphPad) and normalized against untreated (100%) and uninfected (0%) samples to generate drug curves and IC_50_ values.

### Production of recombinant PfGSK3

*Pf*GSK3 was expressed and purified as a GST fusion protein using pGEX-4-T3 in *E. coli* BL21 C41^34^. To remove the GST-tag, the recombinant protein was incubated with Thrombin overnight at 4 °C and loaded directly onto a GSTrap 5 mL column (GE Healthcare). *Pf*GSK3 from the flow through was collected and concentrated.

### *In vitro* phosphorylation assay

Parasite lysates were prepared as described^53^. For pre-phosphorylation, GST fusion proteins were incubated with 1% bovine PKA and kinase buffer (20 mM Tris pH 7.4; 1 mM DTT; 20 mM MgCl_2_; 2 mM MnCl_2_, 50 mM NaV, NaF, β-glycerophosphate; EDTA-free complete protease inhibitor cocktail (Roche)) with 200 μM ATP and incubated overnight. The fusion proteins were washed 3 times with ice-cold TBS and incubated again with 1% bovine PKA for 3 h followed by another 3 washes. Phosphorylation assays were performed in 50 μL total volume containing kinase buffer, 1 μCi γ-^[32]^P-ATP (Perkin Elmer) and 2 μM cAMP or 1 μM CaCl_2_ (where indicated). Reactions were initiated by addition of 3 μg/100 μL total parasite lysate, 1% bovine PKA, 4 μg/mL human GSK3β (Biaffin) or 25 μg/mL recombinant *Pf*GSK3. The samples were incubated for 45 min at 30 °C, stopped by the addition of Laemmli sample buffer, and analysed by SDS-PAGE. If Thrombin cleavage was necessary, beads were washed three times with ice-cold PBS and GST-tag was cleaved by addition of 0.04 U biotinylated Thrombin according to manufacturer’s protocol (Novagen). SDS-PAGE gels were analysed for total protein by staining with coomassie, and by radiolabeling using a Fuji FLA-7000 or a Typhoon phosphorimager. Two independent experiments were performed in triplicate. To establish background levels of phosphorylation, the density of the GST autoradiograph signal was subtracted from the GST-AMA1 signals. This set the GST sample to 0% and AMA1_WT_ protein was normalised to 100%.

### *In vivo* phosphorylation assay

Compounds were prepared in DMSO and added to late-stage parasite cultures (44–48 hour post invasion) at a final concentration of 0.2% DMSO then incubated for 2 h at 37 °C. Sample dephosphorylation was carried out with 400U Lambda phosphatase (New England Biolabs) according to the manufacturer’s protocol. Nunc MaxiSorp 96 well ELISA plates were coated with 10 μg/mL mouse anti-*Pf*AMA1 (1F9) (Walter and Eliza Hall Antibody Facility) or rabbit anti-*Pf*AMA1 (Hodder *et al*., 2001) and blocked with 1% BSA. Treated parasites were lysed and added to the wells to purify AMA1 (4 °C, 16 h). After washing with TBS, the wells were probed with 1 μg/mL rabbit anti-*Pf*AMA1S610p (Genscript [peptide: SFWGEEKRASpHTTPV]) or mouse anti-phosphothreonine (Abcam). The rabbit anti-*Pf*AMA1S610p was detected with 0.2 μg/mL goat anti-rabbit antibody conjugated to horseradish peroxidase (Abcam), and the mouse anti-phosphothreonine antibody was detected with 0.2 μg/mL goat anti-mouse antibody conjugated with biotin followed by streptavidin conjugated with horseradish peroxidase (Rockland Immunochemicals). The wells were developed using tetramethylbenzidine substrate (Thermo Scientific) followed by an equal volume of 2 M HCl, and readouts were performed in a spectrophotometer at 450 nm.

### Imaging

Images of unfixed GFP-expressing parasites were captured using a Zeiss Axioskop 2plus microscope with a Hamamatsu Digital camera (Model C4742-95, Zeiss axiovision) with 1 μg/mL DAPI (Roche) for nuclei stain. Confocal microscopy was performed using the Olympus FV1000 confocal microscope. For 3D reconstitution 20–32 z-stacks (0.3 μm step size) were collected using 488 nm laser. All confocal images were analyzed and processed in Imaris 6.2.0. Gauss filters were used with the filter width suggested by Imaris. Single images were processed in Adobe Photoshop CS4.

## Additional Information

**How to cite this article**: Prinz, B. *et al*. Hierarchical phosphorylation of apical membrane antigen 1 is required for efficient red blood cell invasion by malaria parasites. *Sci. Rep.* **6**, 34479; doi: 10.1038/srep34479 (2016).
